
JOURNAL INFORMATION
==============================
NLM Title Abbreviation: ScientificWorldJournal
Journal ID: ScientificWorldJournal
NLM Journal ID: 101131163
Journal ID: TSWJ

The Scientific World Journal

ISSN: 2356-6140
EISSN: 1537-744X
Publisher: Wiley

ARTICLE INFORMATION
==============================
PMCID: PMC5763936
PMID: 21057726
DOI: 10.1100/tsw.2010.209
Article ID: 672487
Article version: 1
Subjects: Clinical Image

Palpebral Involvement as a Presenting and Sole Manifestation of Discoid Lupus Erythematosus

Yaghoobi Reza 1
Feily Amir 1 *
Behrooz Bahar 1
Yaghoobi Elena 1
Mokhtarzadeh Shabnam 2
1 Department of Dermatology, Jundishapur University of Medical Sciences, Ahvaz, Iran
2 Department of Dermatological Sciences, University of Florence, Italy
*Amir Feily: dr.feily@yahoo.comAcademic Editor: Daniel Batlle

Publication date: 2010
Electronic publication date: 2010 Nov 4
Volume: 10
First page: 2130
Last page: 2131
Received 2010 Aug 27; Revised 2010 Oct 13; Accepted 2010 Oct 14
Copyright: Copyright © 2010 Reza Yaghoobi et al.
Copyright year: 2010
License: This is an open access article distributed under the Creative Commons Attribution License, which permits unrestricted use, distribution, and reproduction in any medium, provided the original work is properly cited.
License URL: https://creativecommons.org/licenses/by/3.0/

Keywords: palpebral involvement, DLE

==============================
Clinical Image
*TheScientificWorldJOURNAL* (2010) 10, 2130–2131
ISSN 1537-744X; DOI 10.1100/tsw.2010.209

TheScientificWorldJOURNAL
www.thescientificworld.com

### Palpebral Involvement as a Presenting and Sole Manifestation of Discoid Lupus Erythematosus

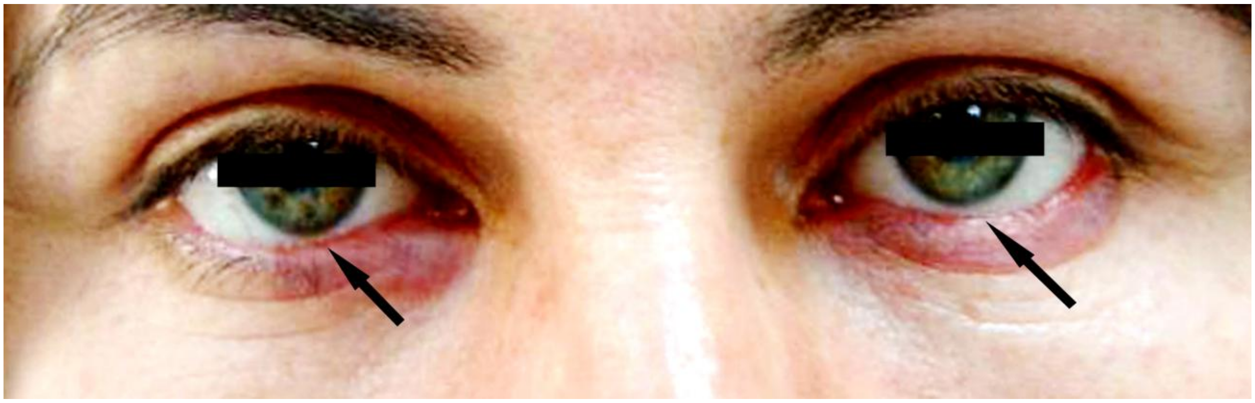

**FIGURE 1**. Edematous, erythematous plaques of the lower eyelids with madarosis (arrows).

Reza Yaghoobi[1], Amir Feily[1,]*, Bahar Behrooz[1], Elena Yaghoobi, and Shabnam Mokhtarzadeh[2]

*[1]Department of Dermatology, Jundishapur University of Medical Sciences, Ahvaz, Iran; [2]Department of Dermatological Sciences, University of Florence, Italy*

**E-mail**: Yaghoobi_rz@yahoo.com; dr.feily@yahoo.com; bahar_61558@yahoo.com; elenayaghoobi@yahoo.com; shabmz@yahoo.it

*Received August 27, 2010; Revised October 13, 2010; Accepted October 14, 2010; Published November 4, 2010*

**KEYWORDS**: DLE, palpebral involvement

A 28-year-old woman presented with a 2-year history of idiopathic, chronic blepharitis unresponsive to several courses treatment of corticosteroid eye drops. Physical examination was notable for edematous, erythematous plaques of the lower eyelids with madarosis in the absence of preceding skin scarring (Fig. 1). Biopsy specimen was obtained and diagnosis of discoid lupus erythematosus (DLE) was made. DLE is a chronic, cutaneous disease that is clinically characterized by a malar rash, acute erythema, and discoid lesions[1]. Localized DLE occurs when the head and neck only are affected, while widespread DLE occurs when other areas are affected, regardless of whether disease of the head and neck is seen[2,3,4,5,6]. Patients with widespread involvement often have hematologic and serologic abnormalities, are more likely to develop systemic lupus erythematosus, and are more difficult to treat[7]. A number of skin diseases may be confused with DLE, such as psoriasis, seborrheic dermatitis, acne,

*Corresponding author.
©2010 with author.
Published by TheScientificWorld; www.thescientificworld.com rosacea, lupus vulgaris, sarcoidosis, Bowen's disease, polymorphous light eruption, lichen planopilaris[8], dermatomyositis[9], granuloma annulare[10], and granuloma faciale[11]. Palpebral lesions may rarely be the presenting or sole manifestation of the disease[12] and lower eyelid involvement is seen in 6% of patients with chronic, cutaneous lupus erythematosus[1]. DLE should therefore be considered as a differential diagnosis in chronic blepharitis or madarosis that persists despite usual medical management and eyelid hygiene[12]. The patient was treated successfully with hydroxychloroquine. The skin lesions resolved with minimal scarring.

### REFERENCES

1. Pianigiani, E., Andreassi, A., De Aloe, G., Rubegni, P., Rufa, A., and Motolese, E. (2002) Chronic erythematous desquamative plaques of the eyelids: discoid lupus erythematosis (DLE). *Arch. Dermatol.* **138(4),** 527–532.
2. Uy, H.S., Pineda, R., 2nd, Shore, J.W., Polcharoen, W., Jakobiec, F.A., and Foster, C.S. (1999) Hypertrophic discoid lupus erythematosus of the conjunctiva. *Am. J. Ophthalmol.* **127(5),** 604–605.
3. Acharya, N., Pineda, R., 2nd, Uy, H.S., and Foster, C.S. (2005) Discoid lupus erythematosus masquerading as chronic blepharoconjunctivitis. *Ophthalmology* **112(5),** e19–23.
4. Gloor, P., Kim, M., McNiff, J.M., and Wolfley, D. (1997) Discoid lupus erythematosus presenting as asymmetric posterior blepharitis. *Am. J. Ophthalmol*. **124(5),** 707–709.
5. Ena, P., Pinna, A., and Carta, F. (2006) Discoid lupus erythematosus of the eyelids associated with staphylococcal blepharitis and Meibomian gland dysfunction. *Clin. Exp. Dermatol.* **31(1),** 77–79.
6. Huey, C., Jakobiec, F.A., Iwamoto, T., Kennedy, R., Farmer, E.R., and Green, W.R. (1983) Discoid lupus erythematosus of the eyelids. *Ophthalmology* **90(12),** 1389–1398.
7. Frith, P., Burge, S.M., Millard, P.R., and Wojnarowska, F. (1990) External ocular findings in lupus erythematosus: a clinical and immunopathological study. *Br. J. Ophthalmol*. **74(3),** 163–167.
8. http://www.wrongdiagnosis.com/d/discoid_lupus_erythematosus/misdiag.htm (last accessed on October 13, 2010)
9. http://emedicine.medscape.com/article/1064945-overview (last accessed on October 7, 2010)
10. http://emedicine.medscape.com/article/1123031-overview (last accessed on October 7, 2010)
11. http://emedicine.medscape.com/article/1083474-overview (last accessed on October 7, 2010)
12. Selva, D., Chen, C.S., James, C.L., and Huilgol, S.C. (2003) Discoid lupus erythematosus presenting as madarosis. *Am. J. Ophthalmol*. **136(3),** 545–546.

**This article should be cited as follows:**

Yaghoobi, R., Feily, A., Behrooz, B., Yaghoobi, E., and Mokhtarzadeh, S. (2010) Palpebral involvement as a presenting and sole manifestation of discoid lupus erythematosus. *TheScientificWorldJOURNAL* **10**, 2130–2131. DOI 10.1100/tsw.2010.209.
